# Neutrophil-to-lymphocyte ratio and platelet-to-lymphocyte ratio are early predictors of bronchopulmonary dysplasia

**DOI:** 10.1097/MD.0000000000034987

**Published:** 2023-09-01

**Authors:** Junsheng Jiang, Yueyan Mao, Qian Zhou, Jiabo Wu

**Affiliations:** a Department of Pediatrics, Linping Branch, the Second Affiliated Hospital of Zhejiang University, Hangzhou, China; b Department of Emergency, The Second Affiliated Hospital of Zhejiang University, Hangzhou, China.

**Keywords:** bronchopulmonary dysplasia, neutrophil-to-lymphocyte ratio, platelet-to-lymphocyte ratio, preterm infants

## Abstract

To determine whether neutrophil-to-lymphocyte ratio (NLR) and platelet-to-lymphocyte ratio (PLR) are correlated with bronchopulmonary dysplasia (BPD) on the first day of prematurity and to help with early warning, identification, and intervention in the development of BPD. From January 2017 to June 2022, newborns who were diagnosed with BPD conducted a retrospective cohort study. Complete blood cells were measured within the first 24 hours of life in preterm neonates of 32 gestational weeks with BPD as the observation group and non-BPD infants as the control group. In all groups, the NLR and PLR levels were measured. Both univariate and multivariate logistic regression analyses were used to evaluate the data. In this research 76 cases of non-BPD and 48 cases of BPD were used as controls. Compared with the non-BPD group, the NLR and PLR levels were considerably higher in the BPD group. Logistic regression analysis suggested that NLR and PLR were independent risk factors for BPD (OR [odds ratio]: 3.786; 95% CI [confidence interval]: 1.75–8.16; *P* < .05; OR: 3.391; 95% CI: 1.85–28.78; *P* < .05). The findings may demonstrate that higher NLR and PLR are independently and significantly associated with the development of BPD.

## 1. Introduction

Bronchopulmonary dysplasia (BPD) is the most common chronic lung disease in preterm infants.^[1]^ In recent years, due to advances in perinatal medicine, the use of pulmonary surfactants and ventilators, the birth and survival rates of very low birth weight infants have significantly increased. Meanwhile, the incidence of BPD has been increasing year by year,^[2]^ seriously impacting the survival and prognosis of preterm infants. However, there is still a lack of effective measures for preventing and treating BPD.^[3,4]^ Therefore, identifying markers to predict the risk of BPD and exploring the pathogenesis of BPD can help in the clinical prevention, diagnosis, and treatment of BPD at an earlier stage, therefore improving the prognosis and quality of survival of preterm infants. The blood count is widely used in clinical practice and its measurement is a routine examination for all patients at hospital admission. It has received growing attention in recent years, because of its advantages of being easily accessible and requiring no additional trauma to the child. Among them, neutrophil-to-lymphocyte ratio (NLR) and platelet-to-lymphocyte ratio (PLR) are new evaluation indicators of systemic inflammation, which have been proven to have diagnostic and prognostic value in the respiratory system and a variety of inflammatory diseases.^[5,6]^ However, studies on the correlation between NLR, PLR, and BPD in preterm infants after birth remains unclear. Therefore, this study was performed to analyze the relationship between PLR, NLR, and BPD after birth in preterm infants admitted to our hospital at gestational age <32 weeks, to provide early help in the diagnosis and treatment of BPD and thus improve the prognosis.

## 2. Research subjects and methods

### 2.1. Research subjects

Premature infants with a gestational age <32 weeks who were admitted to our hospital neonatal medicine unit from January 2017 to June 2022 were selected for retrospective analysis. The exclusion criteria were: admission at more than 1 day of age, death or abandonment of treatment within 4 weeks of birth, infants who were born with hematologic diseases, congenital anomalies or genetic metabolic abnormalities, and neonatal sepsis and severe asphyxia. The diagnostic criterion for BPD, namely the need for oxygenation for ≥28 days, was based on the diagnostic and grading criteria for BPD developed by the National Institute of Child Health and Human Development in 2000.^[7]^ Children <32 weeks of gestational age were assessed at 36 weeks of corrected gestational age. This study was approved by the medical ethics committee of the Linping Branch of the Second Affiliated Hospital of Zhejiang University. Parents receive written information about the research and give their written agreement for their children to participate.

### 2.2. Data collection

Maternal age, gestational diabetes mellitus (GDM), small for gestational age, delivery methods, gestational hypertension, Apgar score, sex, neonatal respiratory distress syndrome, birth weight, intraventricular hemorrhage, surfactant treatment, patent ductus arteriosus (PDA), and necrotizing enterocolitis were all gathered from the hospital electronic medical record system. Whole blood testing was collected within the first 24 hours of life. NLR stands for neutrophils divided by lymphocyte count, and PLR stands for platelet count divided by lymphocyte count. Blood tests were performed within 24 hours of birth using samples taken from the newborns’ umbilical veins.

### 2.3. Statistical analysis

Statistical analyses were performed using SPSS for Windows Version 26. Statistical data are expressed as n (%), and the χ^2^ test or Fisher exact test was used for comparison between groups. Normally distributed measures are expressed as mean ± standard deviation, and the *t* test for 2 independent samples was used for comparison between groups. Non-normally distributed measures are expressed as median (interquartile range), and the Kruskal–Wallis rank sum test was used for comparison between groups. In the multifactor logistic regression model, the risk factors with statistical significance in the univariate analysis were selected as covariates, and BPD and non-BPD subgroups were used as dependent variables to analyze the independent risk factors for BPD. A receiver operating characteristic curve (ROC) was used to analyze the predictive value of the NLR and PLR on the first day of life for BPD, to determine the best cutoff point, and to calculate the sensitivity and specificity of NLR and PLR for predicting BPD. Differences were considered statistically significant at *P* < .05

## 3. Results

In total,124 premature infants with gestational age <32 weeks were enrolled during the study period. Of those infants, 48 were diagnosed with BPD, whereas 76 did not have BPD (Table 1). The basic clinical characteristics of the premature infants in the BPD group and non-BPD group are summarized in Table 1. Univariable analysis showed that the BPD group had a higher rate of PDA (45.8% vs 11.8%, *P* < .05) (Table 1). In addition, infants with BPD had lower gestational age (28.7 vs 30.6 weeks, *P* < .05), and birth weight (1222 vs 1684 g, *P* < .05) (Table 1). However, there were no statistically significant differences in terms of gender, small for gestational age, Apgar score, retinopathy of prematurity, neonatal respiratory distress syndrome, intraventricular hemorrhage, necrotizing enterocolitis, and gestational diabetes mellitus (*P* > .05).

**Table 1:** Clinical characteristics by BPD status.

	BPD (n = 48)	No BPD (n = 76)	*P* value
Gestational age, wk	28.7 ± 1.9	30.6 ± 1.4	<.05
Birth weight, g	1222 ± 283	1684 ± 271	<.05
Male, sex	23 (48.0)	43 (56.6)	.37
Vaginal delivery	22 (45.8)	39 (51.3)	.35
SGA	4 (8.3)	13 (17.1)	.17
1 min Apgar score	8 (6–9)	8 (7–9)	.14
5 min Apgar score	9 (8–10)	9 (8–10)	.06
ROP	6 (12.5)	5 (6.6)	.11
PDA NRDS	22 (45.8) 32 (66.6)	9 (11.8) 37 (48.7)	<.05 .21
Pulmonary arterial hypertension	5 (10.5)	3 (3.9)	.28
IVH grade 3 or 4	9 (18.8)	6 (7.9)	.42
Maternal age	30.1 ± 4.0	29.9 ± 5.0	.79
GDM	13 (27.1)	22 (28.9)	.82
Gestational hypertension	5 (10.4)	15 (19.7)	.17
Premature rupture of membranes	25 (52.1)	43 (56.6)	.62
NEC	4 (8.3)	3 (3.9)	.15

Data are presented as mean ± standard deviation, n (%), or median (interquartile range).

BPD = bronchopulmonary dysplasia, GDM = gestational diabetes mellitus, IVH = intraventricular hemorrhage, NEC = necrotizing enterocolitis, NRDS = neonatal respiratory distress syndrome, PDA = patent ductus arteriosus, ROP = retinopathy of prematurity, SGA = small for gestational age.

The comparison of laboratory examinations at birth between infants with and without BPD was displayed in Table 2. The NLR, and PLR were significantly higher in infants with BPD compared with non-BPD infants (1.2 ± 0.9 vs 0.76 ± 0.7, *P* < .05; 65.7 ± 29.4 vs 53.3 ± 22.2 *P* < .05, respectively). The platelet count was significantly lower in infants with than without BPD (213.4 ± 38.8 vs 258.9 ± 43.2 × 109/L, *P* < .05).

**Table 2:** Patients’ laboratory measurements.

	BPD (n = 48)	No BPD (n = 76)	*P* value
PLT,10^9^/L	213.4 ± 38.8	258.9 ± 43.2	<.05
WBC count, 10^9^/L	9.6 ± 2.8	10.2 ± 3.7	.32
Neutrophils count,10^9^/L	4.7 ± 3.3	5.1 ± 3.6	.11
Lymphocyte count,10^9^/L	4.2 ± 2.1	6.1 ± 3.3	.45
NLR	1.2 ± 0.9	0.76 ± 0.7	<.05
PLR	51.2 ± 18.6	42.9 ± 22.8	<.05

Data are presented as mean ± standard deviation.

BPD = bronchopulmonary dysplasia, NLR = neutrophil-to-lymphocyte ratio, PLR = platelet-to-lymphocyte ratio, PLT = platelet count, WBC = white blood cell.

**Figure 1.**
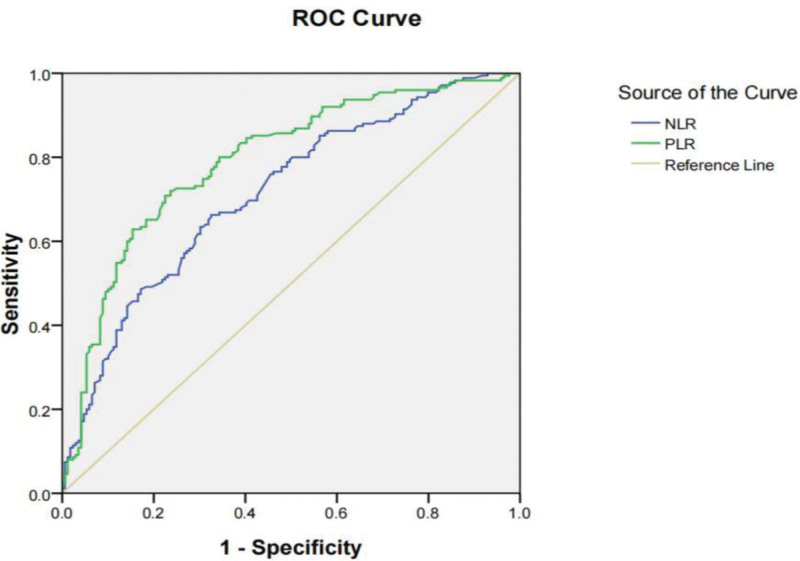
ROC curve analyses of NLR and PLR. NLR = neutrophil-to-lymphocyte ratio, PLR = platelet-to-lymphocyte ratio, ROC = receiver operating characteristic curve.

The logistic regression analysis showed that the NLR (OR [odds ratio]: 3.786; 95% CI [confidence interval]: 1.75–8.16; *P* < .05), PLR (OR: 3.391; 95% CI: 1.85–28.78; *P* < .05), gestational age and birth weight were independent risk factors for BPD, (Table 3). The optimal cutoff value of the NLR was 1.2, with 73.5% sensitivity and 82.6% specificity, and an area under the ROC curve of 0.68 (95% CI: 0.62–0.77), (Fig. 1). The optimal cutoff value of PLR was 58.6, with 74.8% sensitivity and 81.2% specificity, and an area under the ROC curve of 0.78 (95% CI: 0.72–0.83) (Fig. 1).

**Table 3:** Logistic regression analysis showing independent predictors of BPD.

Factors	β	S_b_	Wald	OR	95% CI	*P* value
Gestational age	−0.087	0.056	2.400	0.82	0.56–0.94	<.05
Birth weight	−0.184	0.043	18.21	0.91	0.86–0.98	<.05
PLT	0.494	0.539	0.841	1.64	1.30–1.96	.359
PDA	2.111	1.879	1.262	4.673	2.54–10.40	.261
NLR	1.331	0.392	11.592	3.786	1.75–8.16	<.05
PLR	1.221	0.358	11.632	3.391	1.85–28.78	<.05

CI = confidence interval, NLR = neutrophil-to-lymphocyte ratio, OR = odds ratio, PDA = patent ductus arteriosus, PLR = platelet-to-lymphocyte ratio, PLT = platelet count.

## 4. Discussion

In recent years, the incidence of BPD has increased secondary to the increased morbidity and survival of very early preterm infants. Previous studies have reported that gestational age, birth weight, and PDA are all risk factors for BPD,^[8]^ but the use of NLR and PLR to predict the risk of BPD is less commonly reported. In this study, we analyzed the relationship between NLR, PLR, and BPD in preterm infants on the first day of life and found that NLR and PLR on the first day of life were independent risk factors for BPD and that NLR and PLR after birth were valuable in predicting the development of BPD.

In recent years, it is known that the elevated level of inflammatory cytokines is associated with BPD.^[9,10]^ Premature infants who are exposed to hyperoxia, intrauterine infection, require mechanical ventilation, and develop BPD, have visible infiltration of inflammatory cells and inflammatory factors in their lungs. Within such infiltrations, neutrophils and macrophages play a key role in systemic inflammation in the lung and extra-pulmonary tissues.^[11]^ Compared to non-BPD patients, children with BPD have a higher concentration and greater persistence of inflammatory cells in the bronchoalveolar lavage fluid.^[12]^ These findings demonstrate a link between inflammatory responses and the pathogenesis of BPD.

Generally, Inflammation is usually accompanied by relative changes in the absolute values of circulating peripheral blood leukocyte subpopulations such as neutrophils and lymphocytes. In recent years, leukocyte and their subpopulation counts have been used as markers of the degree of inflammation in several diseases, such as acute appendicitis,^[13]^ allergic rhinitis,^[14]^ chronic obstructive pulmonary disease,^[15]^ and acute pulmonary embolism.^[16]^ Neutrophils are important cells in the immune defense system and regulate the functions of mast cells, epithelial cells, and macro cells.^[17]^ The NLR is a marker of inflammation and is used in conjunction with other inflammatory markers to determine the severity of the body condition in some inflammatory diseases.^[18,19]^NLR was significantly higher in patients with severe pneumonia and acute respiratory distress syndrome compared to controls.^[20]^ In our research, we found that the NLR in the BPD group was higher than that in the non-BPD group at birth. We confirmed that the NLR has the capacity to predict BPD.

Another predictor of systemic inflammation that has been proven to work in some diseases is PLR.^[21,22]^ Recent research has demonstrated the critical role platelets play in angiogenesis, fibrin formation and deposition, platelet parameters, and alterations in premature birth-related diseases, such as sepsis and RDS.^[23,24]^ According to this study, PLR is demonstrated to be associated with the development of BPD, which may be that Platelets and their activation products can participate in the inflammatory response of the body by promoting the aggregation and infiltration of inflammatory cells such as leukocytes and neutrophils.^[25]^

The limited patient population is the primary drawback of this study. Furthermore, because this was a retrospective observational study, it was challenging to ensure consistency of the clinical data because patients were not treated by the same physicians. Finally, this study only included Chinese people, which limits the data available to assess the impact of ethnic diversity. If the level of evidence for the relationship between NLR, PLR, and BPD is to be improved, future studies will need to conduct multi-center, large-sample studies to further validate its association with BPD.

In conclusion, as new inflammatory markers, the NLR and PLR have the advantages of being simple, economical, and rapid, have a certain sensitivity and specificity for predicting the occurrence of BPD, and have important clinical application value.

## Acknowledgment

All authors read and approved the final version of the manuscript.

## Author contributions

**Conceptualization:** Junsheng Jiang.

**Data curation:** Junsheng Jiang.

**Formal analysis:** Junsheng Jiang, Yueyan Mao.

**Funding acquisition:** Junsheng Jiang, Qian Zhou.

**Investigation:** Qian Zhou.

**Project administration:** Qian Zhou, Jiabo Wu.

**Resources:** Qian Zhou, Jiabo Wu.

**Software:** Yueyan Mao, Jiabo Wu.

**Supervision:** Jiabo Wu.

**Validation:** Yueyan Mao.
